# Design and Control of Glycerol-*tert*-Butyl Alcohol Etherification Process

**DOI:** 10.1100/2012/180617

**Published:** 2012-12-05

**Authors:** Elena Vlad, Costin Sorin Bildea, Grigore Bozga

**Affiliations:** Department of Chemical Engineering, University Politehnica of Bucharest, Street Gh. Polizu 1-7, 011061 Bucharest, Romania

## Abstract

Design, economics, and plantwide control of a glycerol-*tert*-butyl alcohol (TBA) etherification plant are presented. The reaction takes place in liquid phase, in a plug flow reactor, using Amberlyst 15 as a catalyst. The products' separation is achieved by two distillation columns where high-purity ethers are obtained and a section involving extractive distillation with 1,4-butanediol as solvent, which separates TBA from the TBA/water azeotrope. Details of design performed in AspenPlus and an economic evaluation of the process are given. Three plantwide control structures are examined using a mass balance model of the plant. The preferred control structure fixes the fresh glycerol flow rate and the ratio glycerol + monoether : TBA at reactor-inlet. The stability and robustness in the operation are checked by rigorous dynamic simulation in AspenDynamics.

## 1. Introduction

As byproduct of biodiesel production, one mole of glycerol (G) is produced for every three moles of methyl esters, which is equivalent to approximately 10% wt. of the total product. As a result of the increased availability, the market price of glycerol has dropped rapidly. Therefore, new uses for glycerol need to be found. Although glycerol could be burnt as a fuel, it could also be processed into more valuable components [1, 2]. 

Di- and triethers of glycerol are compounds soluble in diesel and biodiesel, improving the quality of the fuel [3]. They diminish the emissions of particulate matter, carbon oxide, and carbonyl compounds. Moreover, they provide a 5 K reduction in cloud point and an 8% reduction in viscosity when combined with biodiesel [4]. Therefore, ethers of glycerol are interesting alternatives to commercial oxygenate additives such as MTBE, ETBE, or TAME. Ethers of glycerol can be obtained by etherification with olefins such as i-butene (IB), alcohols such as *tert*-butyl alcohol (TBA) or ethanol [5] or by *trans*-esterification with another ester such as methyl-t-butyl ether. 

Reaction of i-butene with glycerol in presence of homogeneous [6] or heterogeneous [7, 8] acid catalysts yields a mixture of mono-, di-, and tri-*tert*-butyl glycerol ethers (ME, DE, and TE, resp.). Several processes were proposed to perform this transformation [6, 9–12]. In all these processes, the reaction takes place at 14 bar, necessary for keeping the i-butene in liquid phase. Moreover, from the reactor outlet, i-butene is separated as a vapour stream and must be recompressed before being recycled, which is another drawback of the process.

The etherification reaction could be performed at lower pressure using *tert*-butyl alcohol as reactant and ion exchange resins as catalyst [13, 14], according to the following reactions:

(1) $$\begin{array}{c} \text{G}+\text{TBA}⇄\text{ME}+{\text{H}}_{2}\text{O}\\ \text{ME}+\text{TBA}⇄\text{DE}+{\text{H}}_{2}\text{O}\\ \text{DE}+\text{TBA}⇄\text{TE}+{\text{H}}_{2}\text{O} \end{array}$$

Small amounts of i-butene are formed due to TBA dehydration:

(2) $$\begin{array}{c} \text{TBA}⇄\text{IB}+{\text{H}}_{2}\text{O} \end{array}$$

Yusof et al. [15] report glycerol conversions exceeding 64% and mono- to diether ratio in the range 4 : 1–6 : 1, obtained using Amberlyst 15, Amberlite IR-120, Montmorillonite K10, p-toluenesulfonic acid, and sulfuric acid as catalysts. Frusteri et al. [16] studied the etherification of glycerol with *tert*-butyl alcohol in presence of lab-made silica supported acid catalysts. Experiments were carried out in batch mode at temperature ranging from 303 to 363 K. Ozbay et al. [17] compared various solid acid catalysts, such as Amberlyst-15 (A-15), Amberlyst-16 (A-16) and Amberlyst-35 (A-35), Nafion-SAC-13, and gamma-alumina. Amberlyst 15 showed the highest activity at about 110 degrees C, while A-16 gave higher diether selectivity values. Chang and Chen [18] present a systematic optimization of the glycerol etherification with alcohol-*tert*-butylic based on the small-scale experimental data. The reaction conditions (reacting temperature, catalyst loading, solvent loading) leading to maximum glycerol ethers concentration were investigated. Kiatkittipong et al. [19] present several kinetic models for glycerol etherification with TBA, with parameters obtained by regressing measured data from an autoclave reactor. Experiments were performed in a reactive distillation column, the results being compared with AspenPlus simulation predictions. It should be remarked that the column worked as a series of CSTRs (the trays) where the reactants were fed in countercurrent. Therefore, the distillate stream contained a mixture of water and TBA, while the bottom stream contained TBA, glycerol, and mono-, di-, and triether. As a result, separation of reactants and products from the column-outlet streams was still necessary. In a recent paper [20], the liquid phase etherification of glycerol with *tert*-butyl alcohol was investigated in a continuous-flow reactor using Amberlyst-15 as catalyst.

In a different reaction pathway, Al-Lal et al. [21] suggest dehydration to epichlorohydrin followed be etherification with TBA.

Although the feasibility of glycerol etherification with TBA was proved by experimental results, no design, economic evaluation, and controllability analysis of the entire process have been reported. The goal of this work is to fill this gap. The paper is organized as follows. Next section presents the conceptual design of the plant, using a steady state mass-balance model which includes a rigorous model of the chemical reactor and ideal models for the separation section. Following the degree of freedom analysis, plantwide control structures are suggested. For each control structure, the model is solved and the influence of operating parameters on the reactants conversion is analyzed. Thus, a control structure is selected, allowing processing of variable amounts of glycerol and ensuring a unique, steady stable state. Then, detailed design of the separation section is performed and an economic evaluation of the process is accomplished. The performance of the plantwide control (ability to process impure glycerol and to change the production rate) is proved by means of rigorous dynamic simulation performed in AspenDynamics.

## 2. Conceptual Design and Plantwide Control

### 2.1. Reactor-Separation-Recycle Model

**Figure 1:**
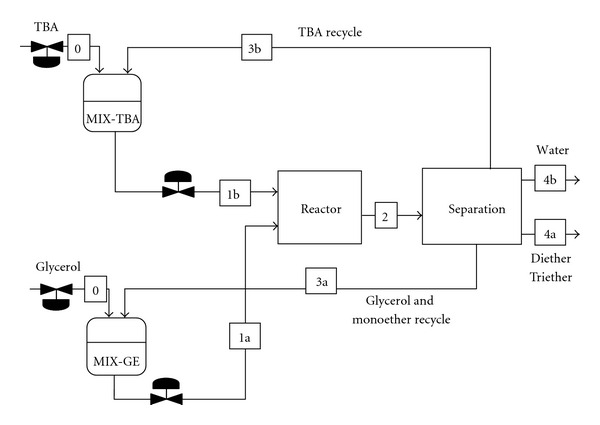
Reactor-separation-recycle structure of the glycerol-TBA etherification plant.

In this section, a simplified model of the plant will be used to assess the feasibility of the process and to perform a preliminary mass balance of the plant. Based on this model, several plantwide control structures will be suggested and evaluated.  Figure 1 presents the Reactor-Separation-Recycle structure of the plant [22]. After the reaction takes place, the reactor effluent enters the separation section. Here, the di-, triethers and water are removed from the plant, while the monoether and unconverted reactants (glycerol and TBA) are recycled to the reactor, after being mixed with fresh reactants. The notations used here will follow Figure 1. *F*_*k*,*j*_ will denote the mole flow rate of species *k* in stream *j*. Subscripts G, TBA, ME, DE, TE, and W will be used for glycerol,* tert*-butyl alcohol, mono-, di-, triethers, and water, respectively. Subscripts 0, 1, 2, 3 and 4 will denote the feed, reactor-inlet, reactor-outlet, recycle, and product streams, respectively. For example, *F*_G,1  _ stands for the flow rate of glycerol at reactor inlet.

The model of the plant assumes a plug-flow reactor (PFR) operated at a fixed temperature (70°C) and perfect separation. The etherification reactions leading to mono-, di-, and triethers were considered, while TBA dehydration was neglected (this assumption will be relaxed when designing the whole plant). It was assumed that TBA, glycerol, and monoether are recovered from the reactor outlet stream and recycled. The model consists of (3) describing the reactor and from (4) to (9) which describe the separation and recycle:

(3) dFjdm=vR,j,  j=G,TBA,ME,DE,TE,W at    m=0    Fj=Fj,1  ,m∈(0,mcat),

(4) $$\begin{array}{c} \left(F_{\text{G},0}+F_{\text{G},2}\right)-F_{\text{G},1}=0, \end{array}$$

(5) $$\begin{array}{c} \left(F_{\text{TBA},0}+{\text{F}}_{\text{TBA,2}}\right)-F_{\text{TBA},1}=0, \end{array}$$

(6) $$\begin{array}{c} F_{\text{ME},1}-F_{\text{ME},2}=0, \end{array}$$

(7) $$\begin{array}{c} F_{\text{DE},1}=0,\;F_{\text{DE},4}=F_{\text{DE},2}, \end{array}$$

(8) $$\begin{array}{c} F_{\text{TE},1}=0,\;F_{\text{TE},4}=F_{\text{TE},2}, \end{array}$$

(9) $$\begin{array}{c} F_{\text{W},1}=0,\;F_{\text{W},4}=F_{\text{W},2}, \end{array}$$

The reaction rates are calculated by (10) to (13), where *ν* is the matrix of stoichiometric coefficients. The reaction rate constants follow Arrhenius temperature dependence with parameters given in Table 1 [19]:

(10) $$\begin{array}{c} v_{R,j}=\sum_{k}\nu_{k,j}r_{k}, \end{array}$$

(11) $$\begin{array}{c} r_{1}=k_{1}\left(x_{\text{G}}x_{\text{TBA}}-\frac{1}{K_{\text{eq},1}}x_{\text{ME}}x_{\text{W}}\right), \end{array}$$

(12) $$\begin{array}{c} r_{2}=k_{2}\left(x_{\text{ME}}x_{\text{TBA}}-\frac{1}{K_{\text{eq},2}}x_{\text{DE}}x_{\text{W}}\right), \end{array}$$

(13) $$\begin{array}{c} r_{3}=k_{3}\left(x_{\text{DE}}x_{\text{TBA}}-\frac{1}{K_{\text{eq},3}}x_{\text{TE}}x_{\text{W}}\right), \end{array}$$

(14) $$\begin{array}{c} r_{4}=k_{4}x_{\text{TBA}}. \end{array}$$

### 2.2. Plantwide Control Structures

The design degrees of freedom represent the variables that must be specified to completely define the process. Their number can be calculated by subtracting the number of equations from the number of variables. The control degrees of freedom are the variables that can be manipulated, namely, the control valves in the process. For most processes, the number of design degrees of freedom is equal to the number of control degrees of freedom [23]. The model of the glycerol etherification plant consists of 15 equations and contains 17 variables (6 reactor-inlet, 6 reactor-outlet, 2 fresh reactants, and 3 product flow rates). Therefore, 2 degrees of freedom must be fulfilled. This is in agreement with Figure 1, where two valves must be used for level control, leaving 2 valves available for manipulating flow rates.

### 2.3. Steady State Behavior

The aim of this section is to investigate the steady state behavior of an etherification plant when different plantwide control structures are applied. We will assume that the nominal flow rate of fresh glycerol is 2.15 kmol/h, which is the typical output of a 15,000 tones/year biodiesel plant.

It should be remarked that the main task of plantwide control system is controlling the inventory of reactants, products, and impurities. Controlling the inventory of reactants within the plant can be performed in two ways [24]: (a) by evaluating, directly or indirectly, the inventory of each reactant and controlling it by feedback using the corresponding fresh feed as manipulated variable; (b) by fixing the fresh feed rate and using the self-regulation property of the mass balance [25]. The latter assumes that the entire amount of reactant brought into the process is converted into products, which are subsequently separated and removed from the plant. Consequently, three control structures will be further considered. Control structure CS1 attempts controlling the inventory of reactants by the use of feedback [24]. Thus, the flow rates of TBA (*F*_1b_ = *F*_TBA,1_) and glycerol + ethers (*F*_1a_ = *F*_G,1_ + *F*_ME,1_) at reactor-inlet are fixed. The amount of reactants in the buffer vessels are used as indirect indications of inventories. Therefore, accumulation or depletion of reactants is avoided by adjusting the fresh reactant feed rates. 

Control structures CS2 and CS3 make use of the self-regulating property of the mass balance [25]. In both control structures the flow rate of fresh glycerol is set to the value *F*_G,0_. Control structures CS2 and CS3 differ by the second flow specification: TBA at reactor inlet (*F*_1b_ = *F*_TBA,1_) and ratio *r*_1_ = *F*_1b_/*F*_1a_, respectively. It should be remarked that, for all control structures, the amount of product obtained equals the amount of glycerol fed in the process, *F*_4a_ = *F*_G,0_.

#### 2.3.1. Control Structure CS1: Glycerol and TBA Inventories Controlled by Feedback

**Figure 2:**
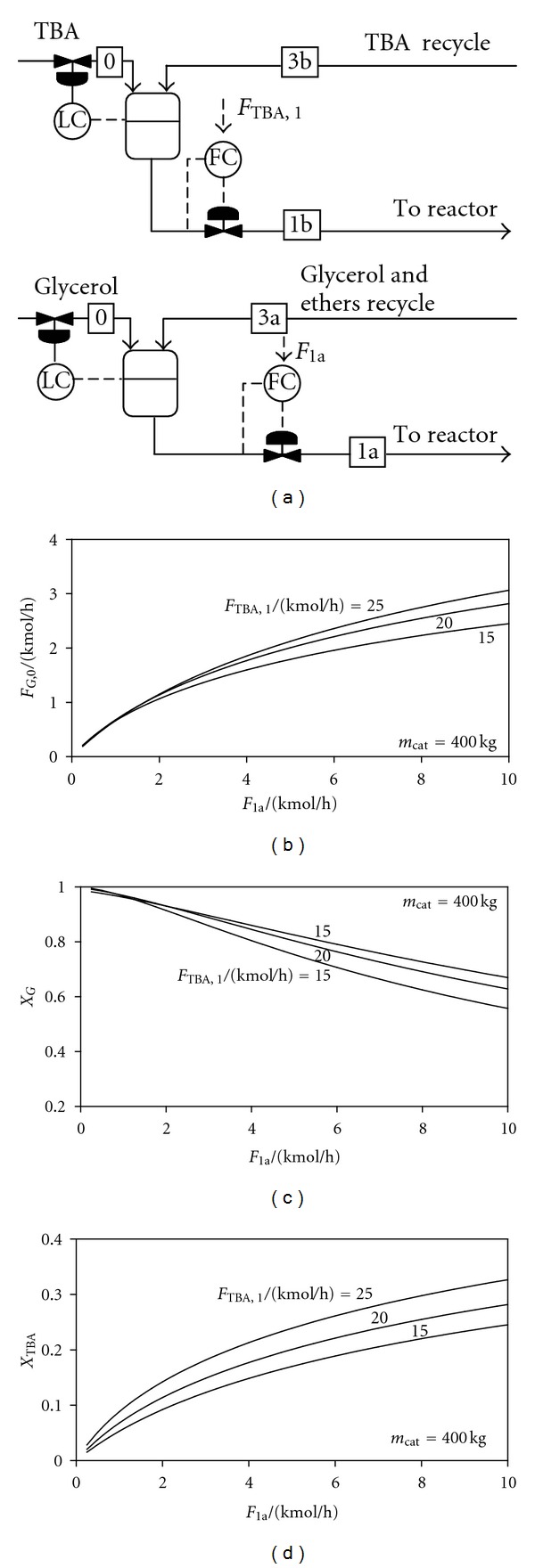
Control structure CS1. Processed glycerol and reactants conversion versus reactor-inlet glycerol + ethers flow rate.

Figure 2 presents the principle of control structure CS1 and results concerning the behavior of the plant when this control structure is applied. The reactor uses 400 kg of catalyst. The top diagram shows the amount of glycerol that is processed (*F*_G,0_) versus the glycerol-ethers reactor-inlet flow rate (*F*_1a_), at different values of the reactor-inlet TBA flow rate (*F*_TBA,1_). It can be observed that the nominal capacity of 2.15 kmol/h could be increased by changing the flow rate *F*_1a_ or by modifying the TBA flow at reactor inlet *F*_TBA,1_. 

The lower diagrams show the dependence of the glycerol and TBA conversions, *X*_G_ = 1 − *F*_G,2_/*F*_G,1_, and *X*_TBA,1_ = 1 − *F*_TBA,2_/*F*_TBA,1_ versus the flow *F*_1a_. For each *F*_1a_ value, a single steady state exists, which is an advantage of this control structure. However, because the fresh glycerol is used to control the buffer-vessel level, this control structure cannot be applied when the flow rate of glycerol is set by the upstream biodiesel plant.

#### 2.3.2. Control Structures CS2-CS3: Self-Regulating Glycerol Inventory

In control structures CS2 and CS3 the flowrate of fresh glycerol *F*_G,0_ is set, which is a very convenient, direct way to change the production rate. In addition, control structure CS2 fixes the reactor-inlet TBA flow rate, while the ratio TBA: glycerol + ME is fixed in CS3.

**Figure 3:**
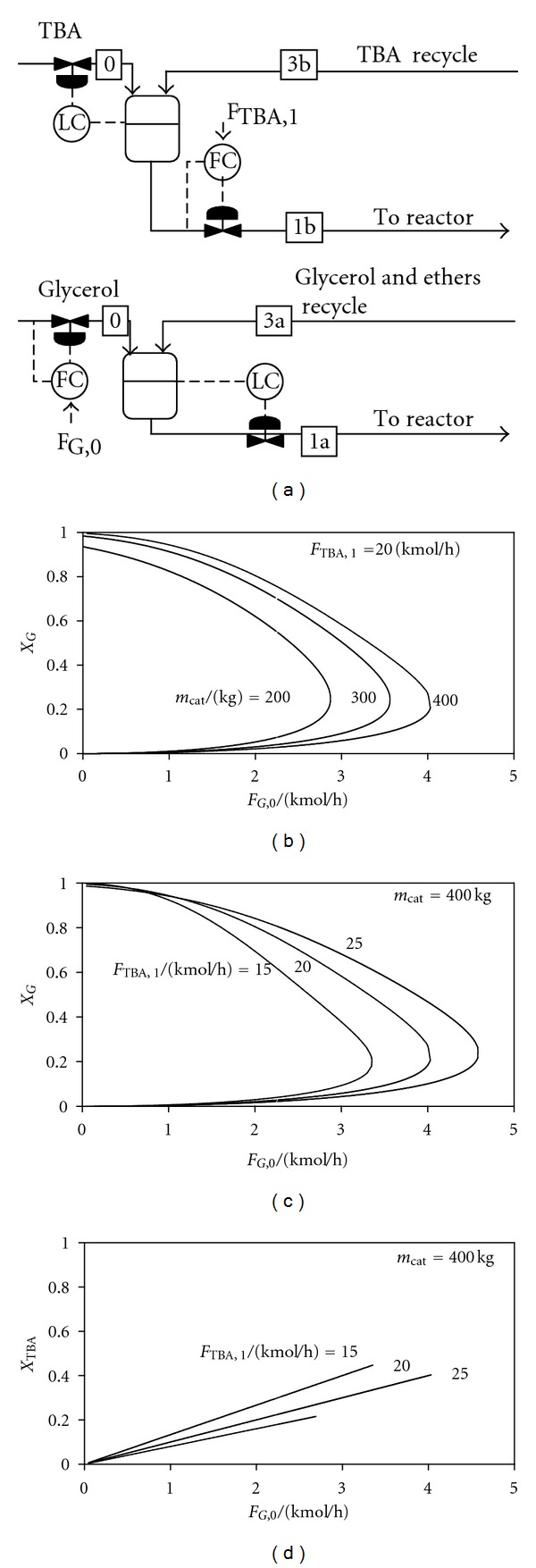
Control structure CS2. Reactants conversions versus fresh glycerol flow rate, for different values of catalyst mass and reactor-inlet TBA flow rate.

Figure 3 presents results obtained when CS2 is applied. The top diagram shows glycerol conversion (*X*_G_) plotted versus the flow rate of fresh glycerol (*F*_G,0_), for different amounts of catalyst used in the reactor. The system exhibits two steady states—at small *F*_G,0_, or no steady state at all—at large *F*_G,0_. This behavior is a major disadvantage of this control structure. It can be observed that an amount of 200 kg of Amberlyst is sufficient to process 2.15 kmol/h of glycerol, but does not allow a large increase of this value. However, 400 kg of catalyst ensures enough flexibility.

The bottom diagrams present the conversion of the glycerol and TBA versus the fresh glycerol flow rate, for different values of reactor-inlet TBA flow rate and 400 kg of catalyst. The extent of the feasibility region increases with the reactor-inlet TBA flow rate. It can be observed that TBA conversion has (almost) the same value on both branches. Moreover, TBA conversion is independent of the amount of catalyst used:

(15) $$\begin{array}{c} X_{\text{TBA}}\approx \frac{2F_{\text{G},0}}{F_{\text{TBA},1}}. \end{array}$$

**Figure 4:**
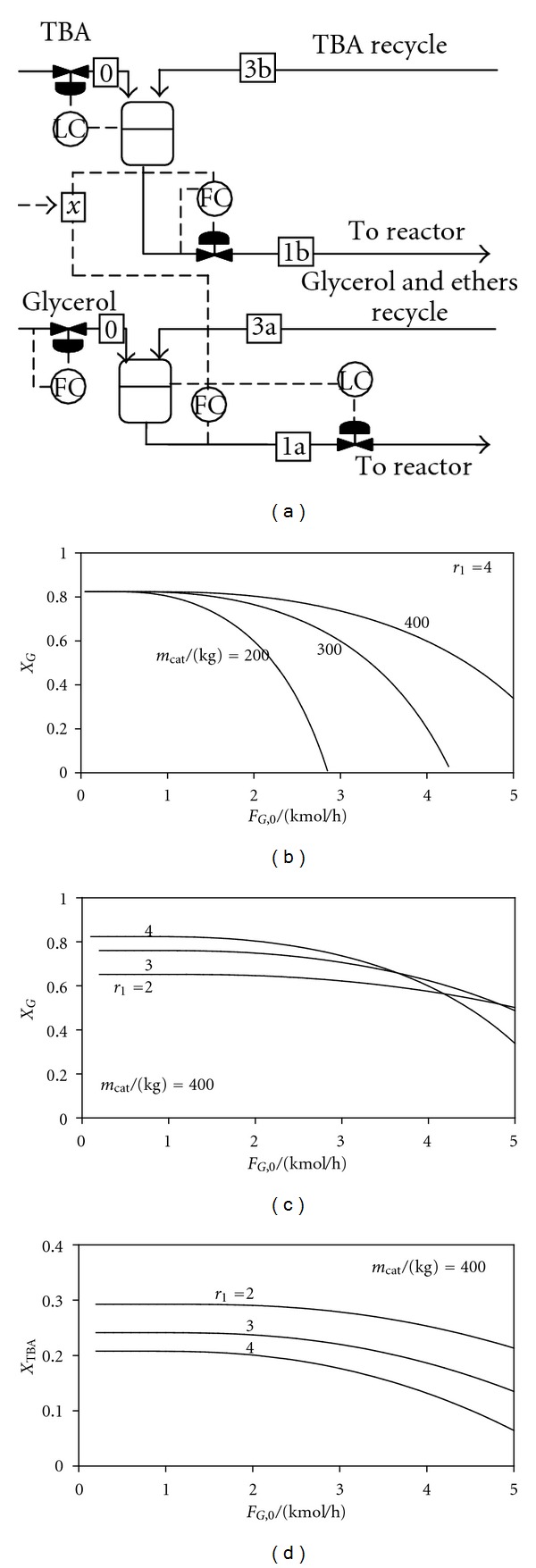
Control structure CS3. Reactants conversions versus fresh glycerol flow rate, for different values of catalyst mass and ratio between reactor-inlet flow rates.

Figure 4 presents results obtained when the control structure CS3 is used. Glycerol conversion versus fresh glycerol flow rate is plotted for different amounts of catalyst. Independently on the catalyst mass and ratio between reactor-inlet flow rates, a unique steady states exists. Compared to CS1, different amounts of glycerol can be processed. However, there is a maximum flow rate of glycerol that can be processed, which increases with the catalyst amount. It can be seen that 400 kg of catalyst allows doubling the production rate. For 400 kg of catalyst, the lower diagrams show that the glycerol and TBA conversions have small sensitivity to the production rate or the ratio between reactor-inlet flowrates.

In conclusion, control structure CS3 offers the advantage of a unique steady state together with easily setting the fresh glycerol flow rate. 

### 2.4. Separation Section

Liquid-liquid and vapor-liquid equilibria were analyzed using AspenPlus. Glycerol and TBA are present in AspenPlus database, from where their physical properties were taken. After defining the molecular structure of the ethers, their properties were estimated using group contribution methods. The behavior of the liquid phase was described by the NRTL activity model. The interaction parameters were taken from Aspen Plus database or were estimated using UNIFAC Dortmund modified method [26].

Table 2 presents the boiling points of the main components and their azeotropes. Small amounts of i-butene that are formed by TBA dehydration can be easily removed due to lower boiling point. The separation of TBA and water from glycerol-ethers mixtures appear to be easy and will be handled by distillation (column C1). 

**Figure 5:**
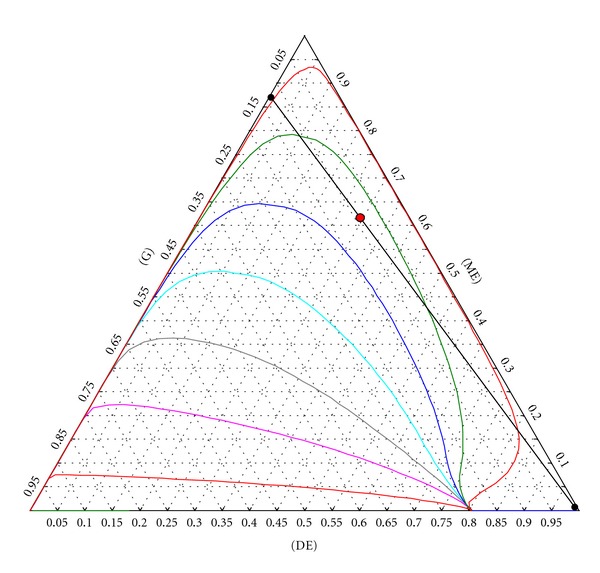
Residue curve map of the glycerol-monoether-diether mixture.

Also, glycerol and monoether which are recycled can be obtained as a bottom product of a distillation column (column C2). Obtaining high purity DE product seems difficult due to the low-boiling G-DE azeotrope. However, the residue curve map (Figure 5) of the DE-ME-G mixture shows only one distillation region where ME acts as a solvent for glycerol, allowing therefore high-purity diether to be obtained in one distillation unit.

TBA and water form a low-boiling homogeneous azeotrope. This can be broken by using a suitable solvent, for example, 1,4-butanediol.

**Figure 6:**
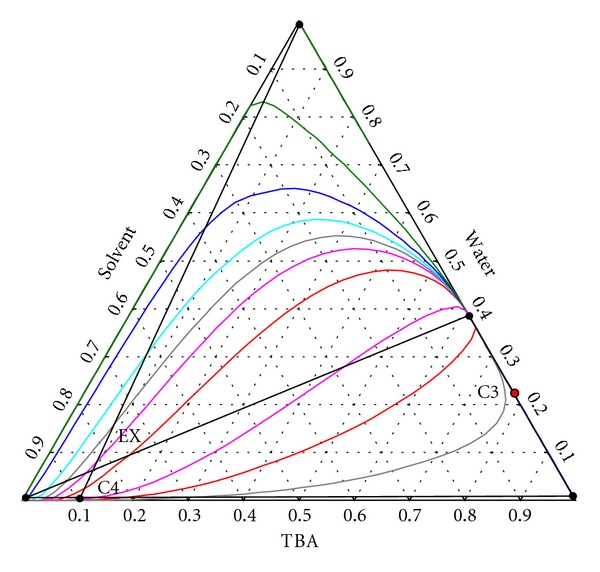
Residue curve map of the TBA-water-1,4-butanediol mixture.

Figure 6 shows the residue curve map of the TBA-Water-1,4-butanediol mixture.

The water-TBA mixture is firstly separated to TBA and azeotrope (column C3). The azeotrope enters in the lower part of the extractive distillation column (EX), while the solvent is fed at the top. The distillate contains water, while the bottom stream consists of solvent and TBA, which is further separated in column C4. 

## 3. Plant Flowsheet

**Figure 7:**
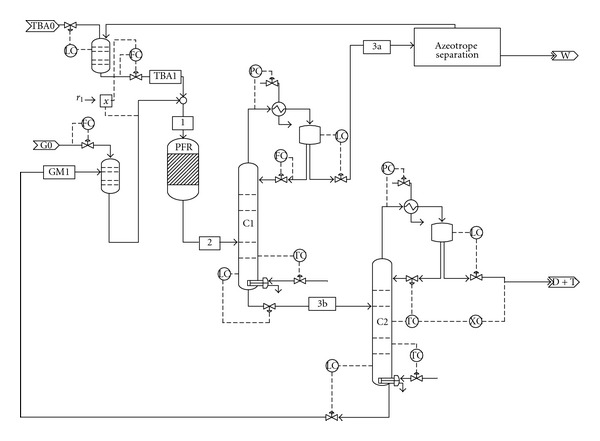
Flowsheet and control loops of the reaction section.

**Figure 8:**
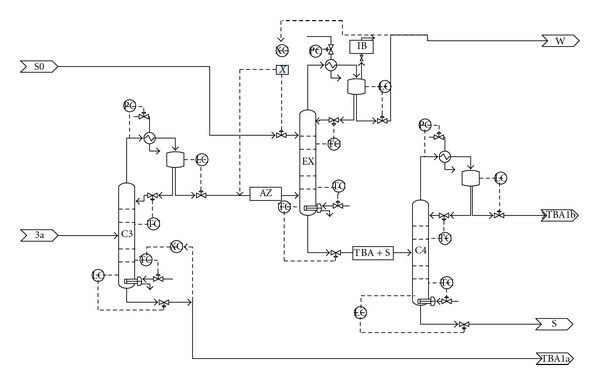
Flowsheet and control loops of the azeotrope separation section.

Figure 7 presents the flowsheet, while Figure 8 details the azeotrope separation section. The control loops are also depicted. A detailed stream report of each section is presented in Tables 3 and 4. The etherification of glycerol with TBA takes place in a plug flow reactor in the presence of 400 kg Amberlyst. The reaction temperature and pressure are set to 70°C and 5 bar, respectively, when the reaction mixture is liquid. The reactor-outlet stream is routed to Column C1. TBA and water are separated as top product, while a mixture of glycerol and ethers leaves the column as bottom product. The column is operated under vacuum (0.1 bar) to avoid high temperature in the bottom of the column. Column C2 separates the mixture of di- and triethers. The bottom product, containing glycerol and monoether, is recycled. The column is operated under vacuum (0.1 bar) to avoid product degradation.

Column C3 separates the TBA/water azeotrope from TBA which is mixed with fresh TBA and recycled. The extractive distillation column (EX) is fed on bottom with TBA/water azeotrope and on top with 1,4-butanediol, which is the solvent. The solvent extracts TBA and is eliminated on the bottom of the column, while the water is removed as liquid on the top. The column has partial condenser in order to eliminate isobutene traces. Column C4 recovers the solvent. TBA is removed on the top of the column, is mixed with TBA stream from column C1 and with fresh TBA and is recycled.

An economic evaluation of the process was performed. A payback period of 10 years was considered and the total annual cost of the plant (TAC) was calculated as the following:

(16) $$\begin{array}{c} \text{TAC}=\frac{\text{capital}\;\text{cost}}{\text{payback}\;\text{period}}+\text{energy}\;\text{cost}. \end{array}$$

The capital cost, including the costs of reactor, distillation columns, and extractive distillation column, was calculated using well-known relationships [27]. The energy cost includes the costs of cooling water (0.08 US$/m^3^) and electricity (8 · 10^−6^ US$/kJ).  Table 5 summarizes the results. 

## 4. Dynamics and Control

The dynamics of the plant must be considered in order to prove the stability of the operating point and the resiliency with respect to disturbances.

For control structure CS3, a dynamic model of the plant was built in AspenDynamics [28]. The controllers were tuned by a simple version of the direct synthesis method. According to this method, the desired closed-loop response for a given input is specified. Then, with the model of the process known, the required form and the tuning of the feedback controller are back-calculated. For all controllers, the acceptable control error, Δ*ε*_max⁡_, and the maximum available control action, Δ*u*_max⁡_, were specified. Then the controller gain, expressed in engineering units, was calculated as *K*_*c*_ = Δ*u*_max⁡_/Δ*ε*_max⁡_ and translated into percentage units. First-order open-loop models were assumed in order to calculate the integral time of the pressure and temperature control loops. As rough evaluations of the process time constants *τ*, 12 min and 20 min were used, respectively. It can be shown that the direct synthesis method requires that the reset time of a PI controller is equal to the time constant of the process, *τ*_*i*_ = *τ*. For the level controllers, a large reset time *τ*_*i*_ = 60 min was chosen as no tight control is required. 

**Figure 9:**
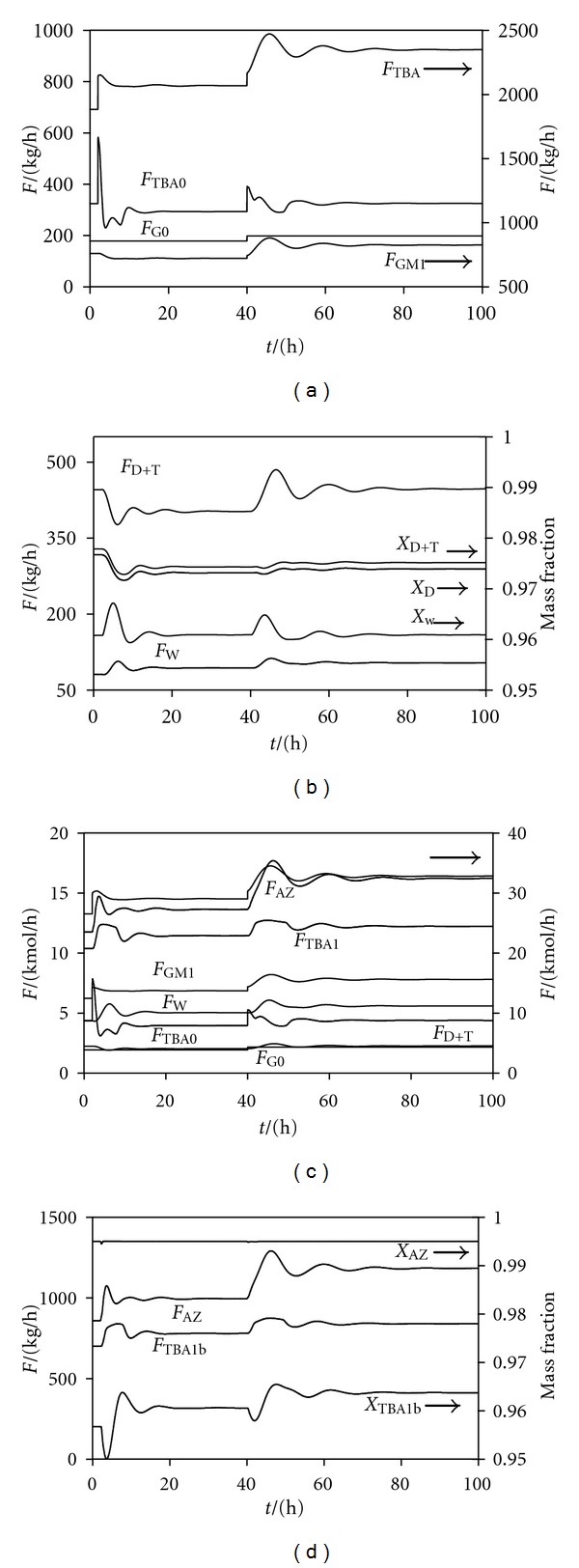
Dynamic simulation results. Refer to Figures 7 and 8 for nomenclature of various streams. *x*_TBA1b_ and *x*_AZ_ are mass fractions of TBA in streams TBA1b and AZ. *x*_D_ is the mass fraction of diether in the product stream D + T.

Figure 9 presents results of dynamic simulation. Molar and mass flow rates together with mass fractions are shown. Starting from the steady state (fresh glycerol: 198 kg/h), two disturbances were introduced. At time of 2 h, a 10% wt. water impurity in the fresh glycerol was introduced. Later (time = 40 h), the flow rate of fresh glycerol (90% wt. purity) was increased to 220 kg/h. It can be seen that the nominal operating point is stable, and the plant achieves stable operation when disturbances are introduced.

## 5. Conclusions

Production of glycerol ethers by etherification of glycerol with *tert*-butyl alcohol catalyzed by heterogeneous acid catalysts, such as Amberlyst 15, is feasible. For a typical glycerol flow rate of 2.15 kmol/h, the reaction can be carried on in a PFR using 400 kg of catalyst. The glycerol conversion is high. However, recycle of the monoether byproduct is necessary. The separation products-unconsumed reactants, are difficult due to formation of the water-TBA azeotrope, which can be broken using a suitable solvent. The TAC of the plant is rather high, 536 000 USD/year. The recommended control structure sets the fresh glycerol feed rate and the ratio G + ME : TBA at reactor inlet.

## Figures and Tables

**Table 1:** Equilibrium constants and rate constants [19].

Equilibrium constant	Rate constant/(mol s^−1^ kg^−1^)
*K*_eq1_ = exp⁡(2.581 − 754.8/*T*)	*k*_1_ = exp⁡(17342 − 6835/*T*)
*K*_eq2_ = exp⁡(1.228 − 942.1/*T*)	*k*_2_ = exp⁡(26953 − 10382/*T*)
*K*_eq3_ = exp⁡(1.779 − 2212/*T*)	*k*_3_ = exp⁡(26953 − 10382/*T*)
—	*k*_4_ = exp⁡(23.358 − 12480/*T*)

**Table 2:** Boiling point for pure components and azeotropes at *P* = 1 bar.

Component/azeotrope	*T*/(°C)	Destination
IB	−6.25	Byproduct
TBA (0.6209)/W (0.3791)	79.97	—
TBA	82.42	Recycle
W	100	Byproduct
G (0.1951)/DE (0.8049)	233.5	—
DE	240.4	Product
ME	256.61	Recycle
G	287.85	Recycle

**Table 3:** Stream results for the reaction section (Figure 7).

Stream name	G0	TBA1	TBA0	GM1	1	2	3a	3b	W	D + T
Flow/(kmol/hr)	2.15	26.5	4.37	6.23	32.75	32.8	26.47	6.32	4.35	2.24
Flow/(kg/hr)	198	1883	324	759	2642	2642	1635	1006.6	80.8	445.6
Temp/(°C)	20	79.8	25	140.9	70	70	29.3	177	25	139.5
Pressure/(bar)	1	5	1	5	5	4.95	1.2	0.15	1	0.1
Flow/(kmol/hr)										
G	2.15	Trace	0	2.94	2.94	0.8	Trace	0.81	0	0.01
TBA	0	24.9	4.37	Trace	24.9	20.6	20.6	0.002	0.04	0.003
ME	0	Trace	0	3.27	3.27	3.27	Trace	3.27	0	0.007
DE	0	Trace	0	0.01	0.01	2.14	Trace	2.14	0	2.13
TE	0	Trace	0	Trace	Trace	0.002	Trace	0.002	0	0.0019
Water	0	1.5	0	Trace	1.5	5.81	5.81	0.005	4.31	0.005
S	0	0.1	0	0	0.1	0.1	Trace	0.1	Trace	0.1
IB	0	0	0	0	Trace	0.04	0.04	Trace	0	Trace

**Table 4:** Stream results for the azeotrope separation section (Figure 8).

Stream name	3a	TBA1a	AZ	S0	W	TBA + S	IB	TBA1b	S
Flow/(kmol/hr)	26.46	11.7	14.7	90.6	4.35	100.9	0.04	10.38	90.5
Flow/(kg/hr)	1635	858.5	776.6	8164.5	80.8	8858	2.22	700.1	8158
Temp./(°C)	29.3	97.8	76	30	25	156	25	80	231
Pressure/(bar)	1.2	1.8	1.0	1.2	1	1.5	1	1.0	1.13
Flow/(kmol/hr)									
TBA	20.61	11.5	9.1	0	0.04	9.05	Trace	9.03	Trace
IB	0.04	Trace	0.04	0	0.003	Trace	0.039	Trace	Trace
S	0	0.0	Trace	90.6	Trace	90.6	0	0.1	90.5
Water	5.8	0.2	5.57	0	4.31	1.2	0.001	1.2	Trace

**Table 5:** Economic evaluation.

Reactor			Column C1	Column C2	Column C3	Column EX	Column C4
Diameter (m)	0.35	Reflux ratio	0.32	1.83	4	1.5	2
		Diameter (m)	0.85	0.55	0.7	0.5	0.55
		No of trays	6	15	41	30	7
Height (m)	2	Reboiler duty (kW)	343	170	716.2	525	563.3
		Condenser duty (kW)	348.7	168.6	654	170	324.4
Cost ($)	9296	Cost ($)	297 966	221 775	589 424	759 704	338 157

Energy Cost = 291 663$/year							
Equipment Cost = 2 440 746$							
TAC = 535 738$/year (10 years payback)							
